# Identification of an Immunogenic Mimic of a Conserved Epitope on the *Plasmodium falciparum* Blood Stage Antigen AMA1 Using Virus-Like Particle (VLP) Peptide Display

**DOI:** 10.1371/journal.pone.0132560

**Published:** 2015-07-06

**Authors:** Erin Crossey, Kathryn Frietze, David L. Narum, David S. Peabody, Bryce Chackerian

**Affiliations:** 1 Department of Molecular Genetics and Microbiology, University of New Mexico, Albuquerque, NM 87131, United States of America; 2 Laboratory of Malaria Immunology and Vaccinology, National Institutes of Allergy and Infectious Diseases, National Institutes of Health, Rockville, MD 20852, United States of America; Tulane University, UNITED STATES

## Abstract

We have developed a peptide display platform based on VLPs of the RNA bacteriophage MS2 that combines the high immunogenicity of VLP display with affinity selection capabilities. Random peptides can be displayed on the VLP surface by genetically inserting sequences into a surface-exposed loop of the viral coat protein. VLP-displayed peptides can then be isolated by selection using antibodies, and the VLP selectants can then be used directly as immunogens. Here, we investigated the ability of this platform to identify mimotopes of a highly conserved conformational epitope present on the *Plasmodium falciparum* blood-stage protein AMA1. Using 4G2, a monoclonal antibody that binds to this epitope and is a potent inhibitor of erythrocyte invasion, we screened three different VLP-peptide libraries and identified specific VLPs that bound strongly to the selecting mAb. We then tested the ability of a handful of selected VLPs to elicit anti-AMA1 antibody responses in mice. Most of the selected VLPs failed to reliably elicit AMA1 specific antibodies. However, one VLP consistently induced antibodies that cross-reacted with AMA1. Surprisingly, this VLP bound to 4G2 more weakly than the other selectants we identified. Taken together, these data demonstrate that VLP-peptide display can identify immunogenic mimics of a complex conformational epitope and illustrate the promise and challenges of this approach.

## Introduction

Virus-like Particles (VLPs) can be used as vaccine platforms to display diverse target antigens in a highly multivalent format that dramatically increases their immunogenicity. Although many specific VLP-based vaccines have been engineered [1], until now VLP technology has not been adapted for use in vaccine discovery. To provide this capability, we engineered the coat protein of MS2, a simple RNA bacteriophage, so that it is highly tolerant of foreign peptide insertions at a site on the VLP surface. We have shown that short (6–10 amino acid) peptide insertions at this site are almost universally (from 80% to >95%, depending on length) compatible with VLP assembly [2–4]. We have constructed large and diverse libraries of VLPs (with >10^10^ individual members) displaying random peptides. Because MS2 VLPs encapsidate the mRNA that encodes coat protein and any guest peptide it carries, we can also perform affinity selections using these libraries. Upon selection with a monoclonal antibody, for example, we can amplify selectant sequences by RT-PCR, regenerate the VLP library, and then repeat this process over multiple rounds of affinity selection.

Previously, we used the MS2 VLP affinity selection system to identify VLPs displaying the linear epitopes of several mAbs [5, 6]. When used to immunize mice, selected VLPs elicited high-titer antibodies that bound to the native target of the mAb. In this study, we asked whether the VLP affinity selection system could also identify epitope mimics (mimotopes) of a more complex conformational epitope and whether these selectants could serve as immunologic mimics and elicit antibody responses against the native target. We hypothesized that the ability to identify epitopes on the same structural platform to be used later in their presentation as a vaccine would increase the likelihood that selected VLPs will be able to elicit antibodies with activities mimicking those of the selecting antibody.

Malaria has been one of the most difficult parasitic pathogens to target with vaccines. The *Plasmodium* parasite encodes more than 5,000 proteins in its genome, many of which are highly variable between strains, and undergoes three separate life stages in humans and the mosquito vector. The parasite is able to infect both hepatocytes and erythrocytes in humans, using different mechanisms for attachment and entry in each case [7, 8]. The clinical manifestations of malaria occur during the blood-stage of the parasite life cycle, after rupture of hepatocytes releases merozoite forms into circulation. Merozoites invade erythrocytes, and mature into trophozoite then schizont parasitic forms, eventually rupturing cells and releasing daughter merozoites [9]. These merozoites can initiate a new cycle of replication in erythrocytes. Several blood-stage protein targets have been identified as candidate vaccine antigens, including apical membrane antigen-1 (AMA1) [10]. Critical functions of this merozoite surface protein in erythrocyte invasion have been described recently, and have shed light on conserved epitopes that may be targeted by vaccines [11, 12]. Although the protein has highly polymorphic and strain-specific regions, it also contains conserved regions that are critical for erythrocyte invasion [13].

Naturally acquired immunity to malaria requires multiple exposures to the parasite over years [14], but immunization studies in animals with recombinant protein as well as screening of infected human sera have revealed that AMA1-specific mAbs capable of neutralizing diverse strains of *Plasmodium* parasites can be elicited [15–17]. Immunization with denatured AMA1 protein fails to elicit parasite-neutralizing Abs, suggesting that invasion-inhibiting antibodies largely target conformational epitopes [15]. Recombinant AMA1 vaccines have been tested in human clinical trials. Although high anti-AMA1 titers can be elicited [18, 19], the immunodominant epitopes on AMA1 are largely strain-specific and vaccination fails to provide protection after multiple malaria seasons [20–22]. While new vaccines containing recombinant protein derived from several *Plasmodium* strains are currently being pursued in animal studies [23], given the propensity of this protein to antigenic variation, multivalent vaccines may also fail to provide universal coverage over time. Alternatively, by specifically targeting conserved regions with critical functions on AMA1 with an epitope-based vaccine, issues of antigenic variation may be avoided. To this end, we have chosen the parasite-neutralizing mAb 4G2 as a candidate to use in our affinity selection strategy.

4G2 is one of the most broadly inhibitory anti-AMA1 monoclonal antibodies that has been identified [24]. 4G2 recognizes a conformational epitope on the conserved face of AMA1 [24–27]. It is likely that 4G2 prevents erythrocyte invasion by blocking AMA1 binding to another merozoite protein (RON2), thereby preventing the formation of the moving junction, a critical intermediate in the invasion process [13]. Indeed, the site of 4G2 binding is in close proximity to a hydrophobic trough critical for this interaction [28]. Attempts to develop vaccines that target the 4G2 epitope by conventional recombinant approaches have largely been unsuccessful [29, 30]. However, by using filamentous phage display, Casey and coworkers identified a peptide mimic of the 4G2 epitope [31], suggesting that an affinity selection approach could potentially be applied to AMA1 vaccine discovery. Here, we show that affinity selection using bacteriophage VLP libraries in combination with deep sequence analysis of selectants readily identifies both individual peptides and peptide families that bind to 4G2. A subset of affinity-selected VLPs elicited antibodies that cross-reacted with AMA1.

## Materials and Methods

### Construction of plasmid libraries

The expression plasmids pDSP62 and pDSP62am have been described previously [2, 5]. Briefly, both plasmids express a single-chain dimer version of the MS2 coat protein. The upstream copy has been ‘codon-juggled’ to allow discrimination of annealing sites by primers during RT and PCR steps. The plasmids contain the phage T7 promoter and terminator regions from the pET3d vector, a kanamycin resistance gene, and an M13 origin of replication. Unique *Sal*I and *BamH*I restriction sites have been engineered upstream and downstream of the insert-containing copy of coat protein, for use in cloning during affinity selection. VLPs produced using pDSP62 contain 90 copies of the displayed peptide per VLP. pDSP62am is similar to pDSP62 except it contains an amber stop codon at the junction of the two coat protein monomers in the single chain dimer. Upon expression [e.g. in the E. coli strain C41(DE3)], the pDSP62am vector produces VLPs that display peptides at low valency (~3 copies of the peptide per VLP) when transformed into C41(DE3) containing pNMsupA, a plasmid expressing an alanine-inserting, amber suppressing tRNA under the control of the *lac* promoter.

We previously constructed random peptide plasmid libraries for use in our VLP affinity selection protocol [5]. Briefly, oligonucleotides were synthesized with 6, 7, 8 or 10 NNS codons, where N represents an equimolar mixture of all four nucleotides and S is an equal mixture of C and G. NNS codons encode all 20 amino acids and only a single stop codon. Using the Kunkel site-directed mutagenesis method and a ssDNA phagemid template, we produced plasmid libraries of at least 10^10^ individual transformants for each peptide library. Plasmid libraries were purified using Qiafilter maxi kits (Qiagen, Venice CA).

### Production of recombinant VLP libraries

Plasmid libraries were electroporated into the *E*. *coli* T7 expression strain C41(DE3) (Lucigen, Middleton WI) and grown to mid-log phase. We were careful to maintain the diversity represented in the plasmid library by accepting only high efficiency of transformation (typically >10^10^ individual transformants) at this step. Coat protein expression was induced by the addition of IPTG (1 mM) for three to five hours and bacteria were collected by centrifugation and the pellet was stored at -20°C overnight. Bacteria were lysed in SCB buffer (50 mM Tris, pH 7.5, 100 mM NaCl) by addition of 10 μg/ml of lysozyme, sonicated and purified from bacterial debris by centrifugation. The supernatant was treated with 10 units/mL of DN*ase*I and the VLPs were purified away from contaminating bacterial proteins by size exclusion chromatography using sepharose CL-4B resin (Sigma-Aldrich, St. Louis MO) as previously described [3]. Fractions that contained VLPs were combined and precipitated by the addition of ammonium sulfate at 50% saturation. Precipitated VLPs were collected by centrifugation, solubilized in SCB buffer and dialyzed in SCB overnight (Slide-a-lyzer cassettes 20K MWCO, Millipore).

### Production of a mutagenic library based on 10-mer selectant

To generate a VLP library based on a selected 10-mer VLP clone, we constructed a mutagenic plasmid library by randomizing the selectant insert nucleotide sequence. Site-directed mutagenesis was performed using ssDNA from the amber stop-containing low-valency plasmid (pDSP62am) as described above. Mutagenic primers were designed through Integrated DNA Technologies as follows. Each of the 30 nucleotide positions encoding the randomized insert in the primer was incorporated with a mix of nucleotides, weighted 76% to the original sequence nucleotide and 8% to each other nucleotide. This library was otherwise expressed and purified as described above.

### Affinity selections

All affinity selections were performed with the mAb 4G2. Briefly, 4G2 was diluted in PBS and adsorbed onto 96-well Immulon 2 plates (ThermoScientific) overnight at 4°C. In the early rounds of selection (typically rounds 1–3), 250ng/well of selecting Ab was used; in the final round of each selection 50ng/well of 4G2 was used. Wells were washed (between each step) five times with PBS. Wells were then blocked for 2 hours at room temperature with 0.5% dry nonfat milk diluted in PBS. VLP libraries were added at 5–10 μg/well in the first round (with equal volumes of 6-, 7-, 8- and 10-mer libraries in the case of the mixed library selection), which was diluted in blocking solution for a total volume of 50uL. In later rounds, 10-25uL/well of ‘crude lysate’ VLPs were used, purified as above but without sepharose column chromatography and with an additional freeze/thaw step followed by centrifugation. Bound VLPs were eluted by applying 50uL of 0.1M glycine, pH 2.7 for 8 minutes, followed by neutralization with 5μL 1M Tris, pH 9.0.

Reverse transcription was performed using 5-10uL of eluate with 2pmol of a primer annealing downstream of the single-chain dimer and MLV reverse transcriptase (Invitrogen) as per manufacturers’ instructions. The DNA product was amplified by PCR using primers that annealed up- and downstream of the insert-containing copy of coat protein. The amplified product was digested using *BamH*I and *Sal*I restriction enzymes, and the resulting product was cloned into either the high- or low-valency plasmid expression vector for additional rounds of affinity selection. In some cases during the selection process individual selectant VLP plasmids were purified and sequenced in order to determine the insert peptide sequence.

### Measuring the relative binding of individual selectant VLPs and libraries

Relative binding of individual selectant VLPs or selectant libraries was determined by capture ELISA. Either 4G2 or isotype mAb (rat IgG2; Jackson ImmunoResearch) was adsorbed onto 96-well Immulon 2 plates (ThermoScientific) at 250ng/well overnight at 4°C. Duplicate wells were washed (between each step) 5 times with PBS. Wells were then blocked for 2 hours at room temperature with 0.5% dry nonfat milk diluted in PBS. VLP libraries from each round of selection, or individual VLP selectants, were diluted in blocking solution and added to both 4G2- and isotype-coated wells at equal concentrations (as determined by denaturing gel electrophoresis) and at several dilutions, for 2 hours at room temperature. Captured VLPs were incubated with rabbit anti-MS2 polyclonal sera for 1 hour at a 1:2,000 dilution in blocking solution, and detected by peroxidase-conjugated goat anti-rabbit IgG antibody (Jackson ImmunoResearch; 1 hour, 1:5,000 dilution). ABTS substrate was used and the reaction was read at OD_405_. OD values of 4G2 mAb wells were normalized to isotype control mAb wells and the ratio was reported.

### Deep sequencing of plasmid libraries

Deep sequencing was performed on intermediate and final round mutagenic and mixed VLP libraries as follows. Selected sequences were amplified from selectant plasmid libraries by PCR, using a bar-coded forward primer and universal reverse primer for each sample. Total PCR product was isolated by agarose gel purification using a Qiagen QIAquick gel extraction kit. Each bar-coded sample was then mixed at equal concentrations and then Ion Torrent (Life Technologies) deep sequencing was performed. Raw nucleotide sequence data was extracted and translated to peptide insert sequences. Peptide selectants were ranked according to number of reads of each unique sequence, and percent representation for each selectant was calculated by dividing the number of individual peptide reads by total reads in the same bar-coded sample. Fold-enrichment for each selectant was calculated by dividing the percent representation of each peptide in the final round by its percent representation in the preceding round.

### Analysis of affinity-selected peptide families

The final round selectants in the mixed library selection were sorted into families using the online Immune Epitope Database analysis resource [32]. Limiting our analysis to the top ~1,450 unique peptide selectants, we set the sequence identity threshold to 80% in the Epitope Cluster Analysis tool and extracted families of at least 10 members. Consensus sequences were ascertained for each family using the online WebLogo resource [33].

### Immunogenicity of recombinant VLP selectants

Individual VLP selectants were isolated and purified as above, or cloned independently using site-directed mutagenesis (also as above) if no colony was picked with the desired peptide insert. VLP formation was confirmed by agarose gel electrophoresis, and VLPs were purified by sepharose chromatography or FPLC then quantified by denaturing gel electrophoresis. For the first immunization scheme, using the 10mer VLP selectant, groups of 3–5 Balb/C mice were given two intramuscular immunizations with either 10 μg selectant VLP or wild type VLP at 2-week intervals, and sera was drawn by intraocular bleed 2 weeks after the second dose. A recombinant AMA1 boost was also given at this time point (AMA1-3D7), and consisted of 25ug protein formulated with 50% IFA. Sera were drawn again 2 weeks after AMA1 immunization. A control group received only the AMA1 vaccination. Anti-AMA1 titers were quantified by sera dilution in a direct ELISA, and anti-peptide titers were quantified by engineering a PP7 bacteriophage to display the 10mer sequence in a similar surface-exposed loop to that of MS2. Briefly 500 ng/well of recombinant AMA1 or recombinant PP7 VLPs were adsorbed to ELISA plate wells overnight at 4°C. Blocking and washing were performed as above, and dilutions of sera were added to wells. Peroxidase-conjugated goat anti-mouse IgG was used for detection of bound sera IgG (1 hour, room temperature), and either ABTS or TMB (neutralized with 1% HCL) was used as substrate. OD was read at 405nm or 450nm, respectively.

In a second immunization scheme, using the mutagenic and mixed library VLP selectants as well as one group receiving the 10mer VLP selectant used previously, groups of four to six week old Balb/c mice were given three intramuscular immunizations of 5μg VLP formulated with 50% incomplete Freund’s adjuvant (IFA; Sigma Aldrich) at 2-week intervals. Sera were taken one week after the final immunization, and anti-AMA1 IgG titers were measured in dilutions of sera by direct ELISA. In some cases IgG titers against AMA1-FVO and AMA1-L32 were also determined. Sera from wild type VLP-immunized animals or from mice prior to immunization served as ELISA controls in all experiments.

All animal studies were carried out in accordance with the recommendation in the Guide for the Care and Use of Laboratory Animals, the Animal Welfare Act, and U.S. federal law. The protocol was approved by the Institutional Animal Care and Use Committee (IACUC) of the University of New Mexico Health Sciences Center.

### 4G2 competition ELISA

A competition ELISA was performed whereby sera from mice immunized with AMA1 only, 10mer selectant VLP only, 10mer VLP-primed/AMA1-boosted or wild type VLP control competed with 4G2 for binding to immobilized AMA1. 4G2-only controls were also included. Briefly, 250ng/well recombinant AMA1 was adsorbed to ELISA plate wells overnight at 4°C. Blocking and washing were performed as above, and dilutions of sera were added to 4G2 (2mg/mL, diluted 1:20,000) in triplicate wells. Peroxidase-conjugated mouse anti-rat IgG was used for specific detection of 4G2 (1 hour, room temperature), and ABTS used as substrate. OD was read at 405nm, and all experimental OD values were normalized to the internal no-sera controls for each experiment so that competition could be compared between separate ELISA runs.

To test whether sera from VLP-vaccinated mice bound to the same epitope as 4G2, the same ELISA protocol described above was performed, except that peroxidase conjugated rat anti-mouse IgG (a mixture of mouse IgG1, IgG2a, and IgG2b specific antibodies, diluted 1:5,000; Zymed) was used for specific detection of mouse IgG bound to AMA1.

### Relative affinity of 4G2 for selectants

For the final mutagenic and mixed library VLP selectants, relative affinity was measured by adsorbing 500ng/well of individual VLPs to ELISA plate wells and incubating overnight at 4°C. Blocking and washing were performed as above, and dilutions of 4G2 were added in triplicate wells. Peroxidase-conjugated mouse anti-rat IgG was used for specific detection of 4G2 (1 hour, room temperature), and ABTS used as substrate. OD was read at 405nm.

### Immunofluorescence assay (IFA)

Mature schizont stage 3D7 parasites were smeared onto slides and fixed with cold methanol for 1 min. After air-drying, the smears were coated with an anti-PfAMA1 monoclonal antibody (1E9; at 10 μg/mL), pre-immune sera (diluted 1:25), or sera from VLP-immunized mice (diluted 1:25) and incubated at room temperature for 1 h. Slides were washed by shaking in PBS for 4 x 5 min then incubated with Alexa 488-conjugated anti-mouse antibody (20 ug/mL) for 1 h at room temperature protected from light. All slides were washed by shaking in 1x PBS for 4 x 5 min then mounted using VECTASHIELD with 1.5 ug/mL DAPI. Fluorescent images were obtained on an Olympus BX51 microscope.

### Structural modeling

The predicted structure of recombinant MS2 coat proteins was determined by the One-to-One Threading function in the Web-based Phyre2 Server [34] by modeling on MS2 coat protein (PDB: 1MSC). AMA1 structures were based on PfAMA1 (PDB:1Z40_A) [25].

## Results

The basic methodology for the VLP affinity selection process has been described previously [5]. Briefly, we constructed four VLP libraries that display random 6-, 7-, 8- or 10-amino acid inserts in an exposed loop structure on the surface of bacteriophage MS2 VLPs. Each library contains more then 10^10^ transformants, and each VLP displays a different guest peptide on its surface and encapsidates its own mRNA. After biopanning using the 4G2 mAb, affinity-selected sequences were recovered by reverse transcription of the coat protein-specific mRNA they contained, followed by polymerase chain reaction. The selected sequences were then re-cloned to produce VLPs for additional rounds of selection. We used three different selection approaches in order to identify mimotopes of the 4G2 epitope, as described below.

### Affinity selection using a 10mer VLP library

Because we were initially concerned that it may be difficult to identify a mimotope of the 4G2 epitope using very short peptides, our first affinity selection utilized our library that contained the longest randomized sequence, 10mers. Four rounds of affinity selection were performed, at increasing stringency. The first two rounds utilized high-valency peptide display on VLPs, in which 90 copies of the random peptide were displayed on each VLP. The final two rounds utilized low-valency display, in which an average of three peptides are displayed per particle (the process by which we make low-valency VLPs is described in more detail in [5]). To further increase stringency in the final round of selection, we used less 4G2 (50 ng) so that VLPs were present at about 50-fold molar excess compared to antibody. Following the fourth round, we picked 24 individual selectants, sequenced them, and then tested for the ability of the selected VLPs to bind to 4G2, but not an isotype control, by ELISA (data not shown). All of the VLPs that exhibited strong binding to 4G2 had the same peptide insert, NWDPTQFPGK. This peptide has no obvious sequence homology to the amino acids that have been implicated in the AMA1 4G2 epitope [11, 12].

**Fig 1:**
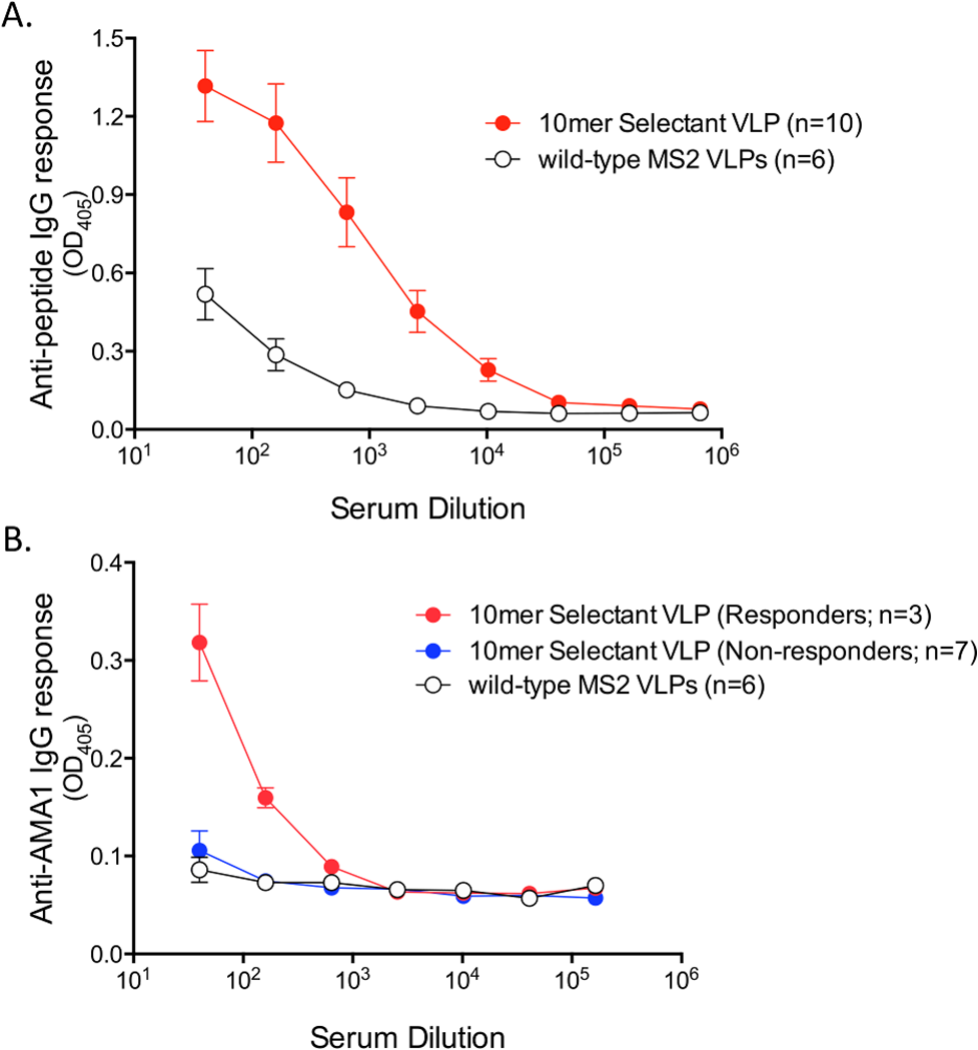
Selectant VLPs elicit high-titer peptide-specific antibody responses but variable AMA1-reactive IgG titers. Groups of mice were immunized twice with 10mer selectant VLPs or wild-type MS2 VLPs. Serum was taken two-weeks following the boost. (A) Vaccine-elicited peptide titers were assessed by ELISA using recombinant PP7 bacteriophage VLPs displaying the NWDPTQFPGK peptide (in a surface-exposed AB loop similar to that of MS2) as the target antigen. (B) Antibody responses against native AMA1. In 3/10 vaccinated mice we observed low-titer cross-reactivity; these are referred to as “responders”. The remaining mice were characterized as “non-responders”. Mean OD_405_ values are shown for each group. Error bars represent standard error of the mean, SEM.

We investigated the immunogenicity of the 10mer selectant (MS2-NWDPTQFPGK) by immunizing a group of ten Balb/C mice with 10 μg of VLPs. Mice were immunized twice, at a two-week interval, and two weeks after the boost peptide-specific and recombinant AMA1-reactive IgG titers were measured by ELISA (Fig 1). To detect peptide-specific responses we constructed a recombinant PP7 bacteriophage VLP that displayed the NWDPTQFPGK peptide and used this VLP as a target antigen in an ELISA. All of the mice immunized with the 10mer selectant VLP had high-titer peptide-specific IgG antibody levels (end point dilution titers of 10^4^−10^5^; Fig 1A). However, only a subset (3 of 10, termed ‘responders’) of the mice immunized with MS2-NWDPTQFPGK VLPs produced antibodies that cross-reacted with recombinant AMA1, although these antibodies were of low titer (Fig 1B). The majority of mice immunized with MS2-NWDPTQFPGK VLPs were ‘non-responders’, in that they failed to elicit AMA1-reactive antibodies. All of the immunized mice, including non-responders, had similarly high peptide titers, so we could not attribute the variable response to gross quantitative differences in the antibody responses in individual mice. We considered the possibility that mice may have a low frequency of B cells that are capable of producing AMA1-reactive antibodies. While this may be the case, the use of higher antigen doses (25 μg) failed to increase the percentage of responders (data not shown).

**Fig 2:**
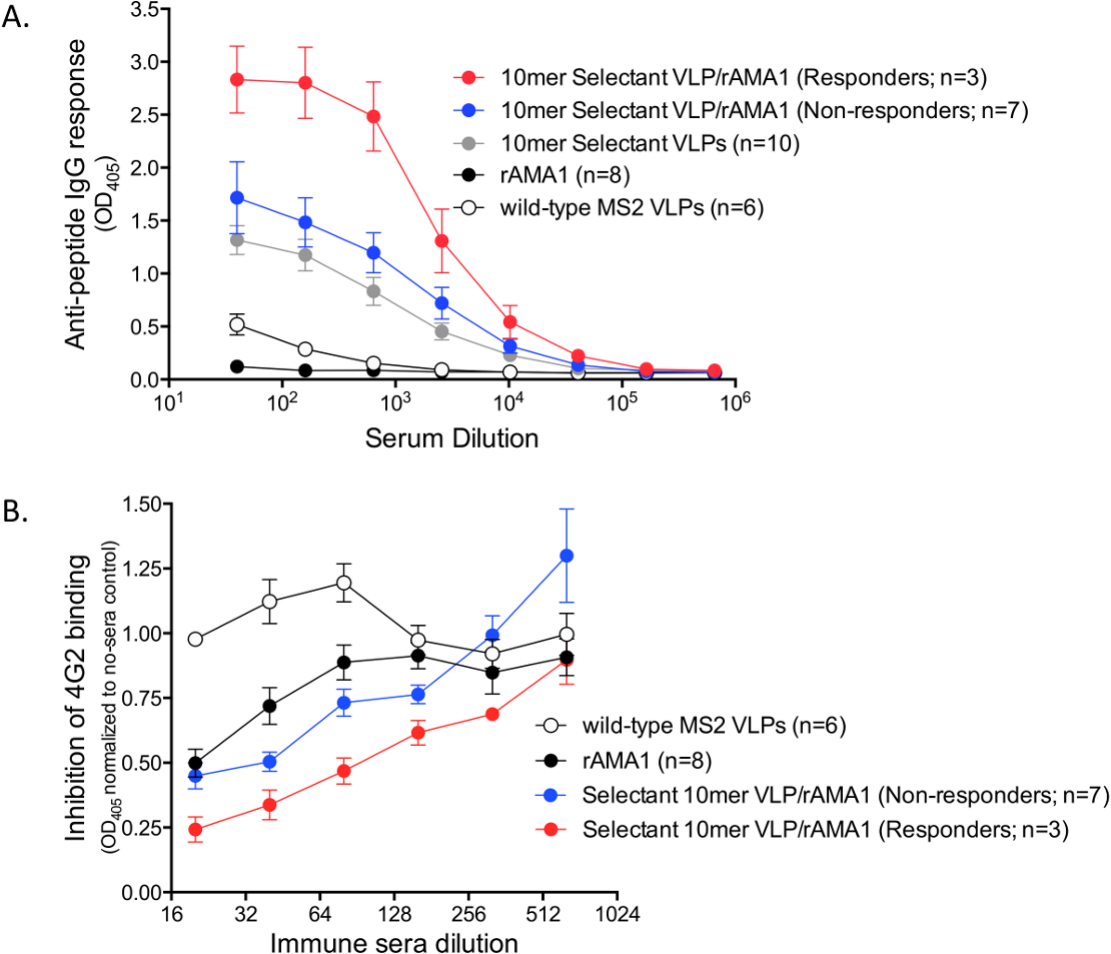
Antibody responses in VLP-immunized mice boosted with recombinant AMA1. (A) Anti-peptide responses in responder mice are enhanced by boosting with 25μg recombinant rAMA1 (with incomplete Freund’s adjuvant). Mice were immunized with selectant VLPs and then boosted with recombinant AMA1. Mice were segregated into responders (red circles) and non-responders (blue circles). Also shown are the anti-peptide responses in mice immunized with selectant VLPs prior to boosting with rAMA1 (grey circles). As controls, groups of mice were immunized with wild-type MS2 VLPs (open circles) or recombinant AMA1 alone (black circles). Immunization with recombinant AMA1 boosts anti-peptide titers, but only in animals that had AMA1-cross-reactive titers present after VLP-selectant immunization. Mean and SEM are shown for each group. (B) Competition of immune sera with 4G2 for AMA1 binding. Sera from immunized mice were incubated with 250 ng rAMA1 on an ELISA plate in the presence of 100ng mAb 4G2. Serum was pooled from mice immunized with selectant VLPs boosted with rAMA1 (responders and non-responders), rAMA1 alone, or, as a control, wild-type MS2 VLPs. Data represent triplicate wells, normalized to the value of the no serum control. Mean OD_405_ and SEM are shown at each dilution.

To better characterize the differences between the responders and non-responders, we boosted MS2-NWDPTQFPGK VLP-immunized mice with 25 μg of recombinant AMA1 (rAMA1) protein and compared antibody responses to mice that were only immunized with rAMA1. Fig 2A shows the subsequent peptide-specific responses in these groups of animals. Immunization with rAMA1 alone elicited antibodies against AMA1 (not shown), but failed to induce antibodies that recognized the 10mer 4G2 mimotope. Boosting non-responder mice with rAMA1 failed to increase anti-peptide IgG titers. However, boosting the responder group with recombinant AMA1 increased the NWDPTQFPGK peptide-specific IgG titers. This provides indirect evidence that immunization with MS2-NWDPTQFPGK VLPs elicited low-titer 4G2-like antibody responses in a subset of animals.

In order to verify this, we tested the ability of immune sera from responders and non-responders boosted with AMA1, or mice immunized with AMA1 alone, to compete with 4G2 for binding to AMA1. As shown in Fig 2B, sera from AMA1-boosted responders had the greatest ability to inhibit 4G2 binding to AMA1. There was a 2.5-fold difference in the sera dilutions capable of inhibiting 50% binding of 4G2 to AMA1 in the AMA1-boosted responders (1:100) as compared to AMA1-boosted non-responders (1:40) and the sera from mice immunized with AMA1 alone (1:20) (Fig 2B).

Selections using mAbs that recognize linear epitopes often yield a single VLP selectant after only a few rounds of selection [5, 6]. In contrast, we had anticipated that selections using a mAb recognizing a conformational epitope would result in the identification of multiple mimotope sequence families. Since only one clone was identified in the 4G2-10mer selection, we were concerned that even with 10^10^ individual clones, our library may have been too small to contain the other members of this sequence family. Therefore, we pursued two additional selection strategies. First, we reasoned that if we had found the only sequence member of a theoretical family of peptide mimotopes for 4G2 that was not represented in the original 10mer library, we could recreate a mutagenized version of this family and repeat the 4G2 selection. In this way we might potentially improve upon the variable immunogenicity of our original 10mer selectant by finding a mutagenized selectant sequence that acted as a better mimotope. Secondly, we asked whether 4G2 mimotopes might be better represented as different-length peptides than 10mers. We therefore also performed 4G2 affinity selection using a mixed VLP library with 6-10mer inserts represented. In each case we performed deep sequencing after each round of selection in order to more carefully monitor the selection process.

### Affinity selection using a mutagenic library based on the 10mer selectant

A library of recombinant VLPs based on the original 10mer selectant was generated by randomizing the nucleotide sequence encoding the peptide insert. This was achieved by mutagenic primer design utilizing weighted mixes of nucleotides at each of the 30 positions (76% original nucleotide and 8% each other nucleotide). Using these methods we generated a VLP library with approximately 10^7^ members. The library was deep sequenced to confirm the mutagenic frequency. The frequency of mutation at each nucleotide position was as anticipated (~24%; S1 Table); ~0.1% of the mutagenic library represented the parental 10mer peptide sequence

**Fig 3:**
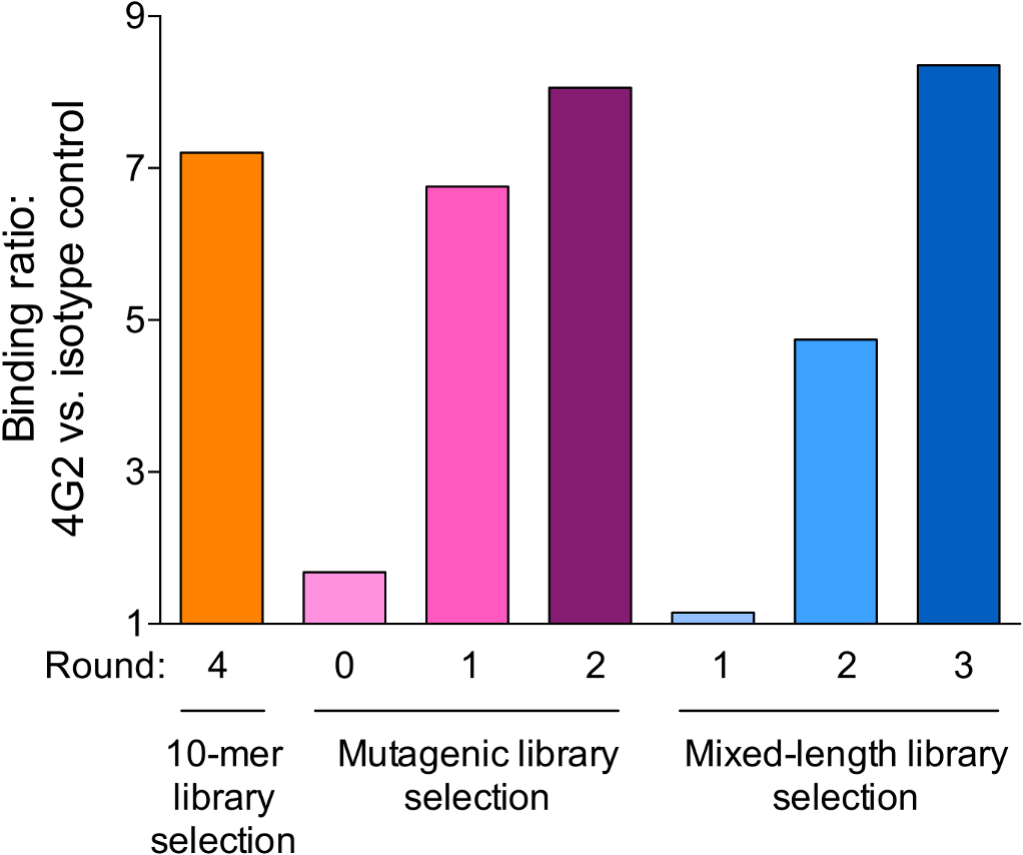
Relative binding of selectant VLPs libraries was monitored by capture ELISA. Pooled crude VLP lysates, in duplicate, were applied to wells coated with 4G2 or an isotype control. VLP binding was detected using rabbit anti-MS2 polyclonal antibody. Relative binding was calculated by using the ratio of mean binding to 4G2 with the isotype control. The orange bar represents the final library from a selection using only a 10-mer insert VLP library (i.e. the library from which NWDPTQFPGT was identified). The pink bars represent all the stages of selection, including the original mutagenic library (round 0), using a VLP library with insert sequences randomized from the 10-mer NWDPTQFPGK selectant. The blue bars show the results of the mixed length VLP library selection. This experiment was repeated three times using different dilutions of the VLP libraries; similar ratios were observed in each experiment.

Because some proportion of this library already had the ability to bind to 4G2, we performed just two rounds of selections using libraries in which the peptides were displayed at low-valency. Selections were monitored by capture ELISA, by comparing ability of 4G2 versus an isotype control to bind to selected VLPs. As shown in Fig 3, a single round of selection using the mutagenic library resulted in substantial enrichment of the 4G2-binding population of VLPs. After each round of selection, VLPs were deep sequenced and the enrichment of specific peptide selectants was calculated (S2 Table). The original 10mer clone remained the most represented by number of reads total in each round. However, several other peptides were strongly enriched during the selection process.

### Affinity selection using a mixed insert-length VLP library

**Fig 4:**
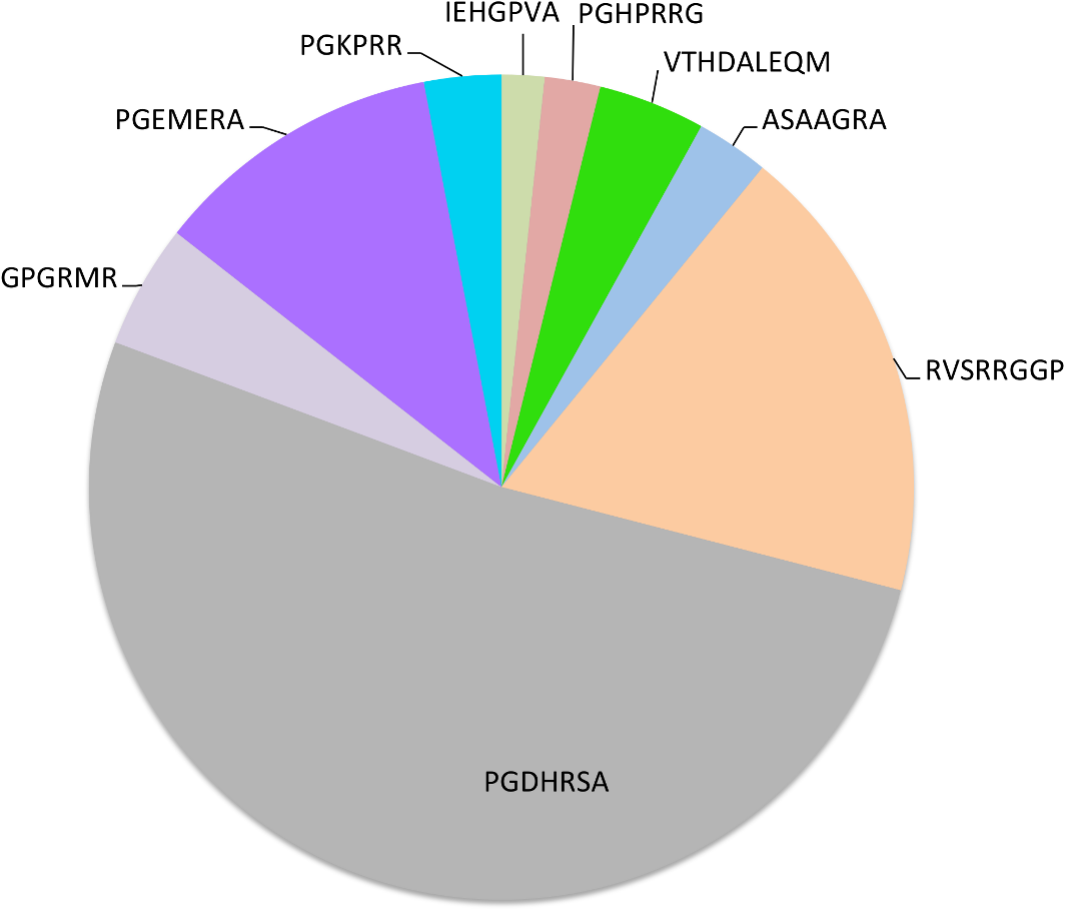
Peptide families identified after 4G2 selection of a mixed VLP library. After 3 rounds of selection VLPs were deep sequenced. The top 1,450 unique peptide selectants were sorted into families using the online Immune Epitope Database analysis resource [32]. This analysis identified 10 peptide families with at least 10 members. This chart shows the consensus sequences and relative frequencies of each of these families.

To address our second concern, that 4G2 mimotopes may be better identified from a more length-diverse library of recombinant VLPs, we performed 3 rounds of affinity selection with a mixed library of VLPs displaying 6-10mer peptides. We performed one round of selection at high valency, and two rounds at low-valency. The enrichment of 4G2 binding in selectant populations is shown in Fig 3. By round 2 the VLP selectant population bound to 4G2 above background levels, and binding activity was enriched after a third round of selection. Deep sequencing of the round 3 selectant population identified ~1,450 unique selectant sequences (summarized in S3 and S4 Tables), and using the Immune Epitope Database analysis resource [32] we identified nine sequence families of more than 10 members each; Fig 4).

### Characterization of the antigenicity and immunogenicity of 4G2 selectants

**Fig 5:**
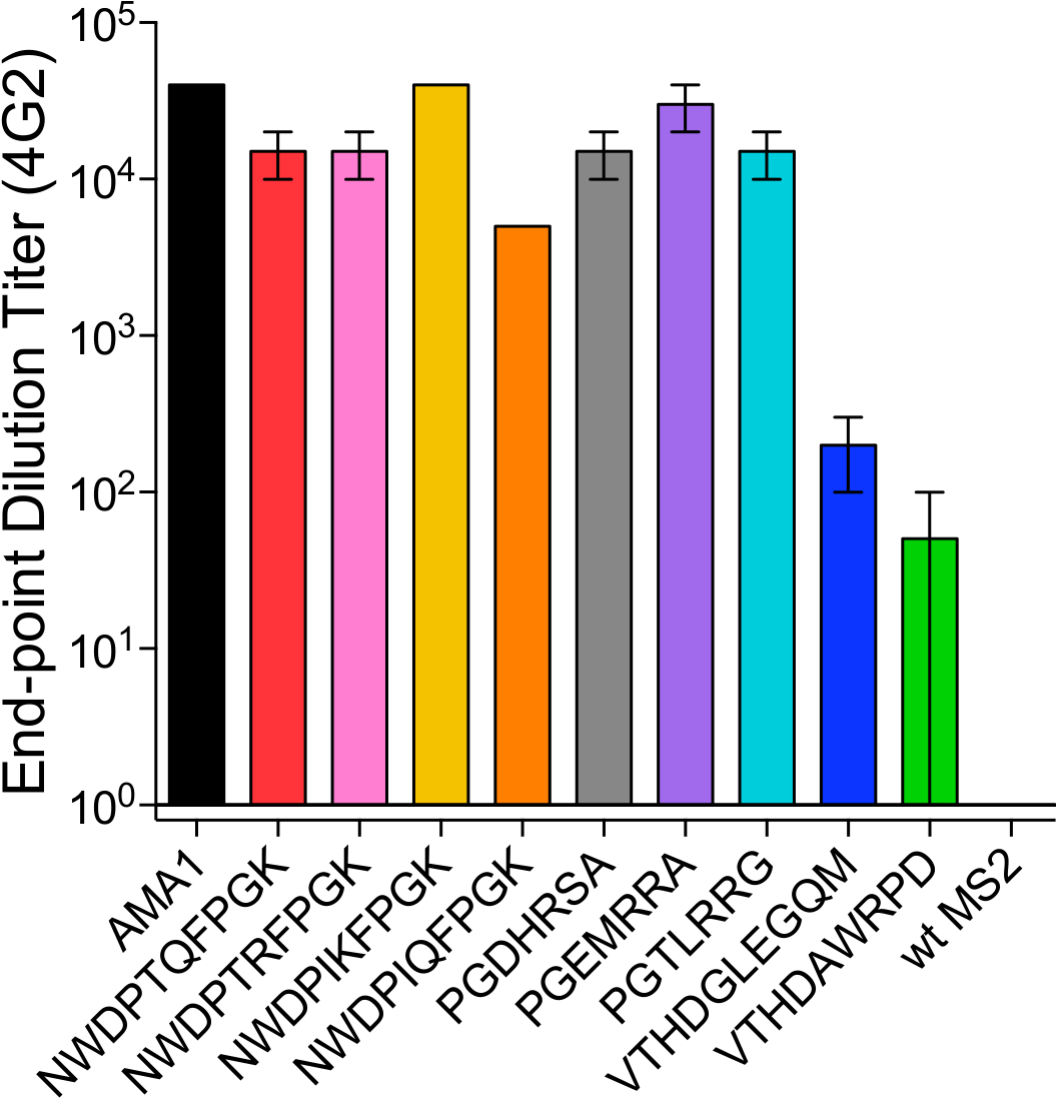
Relative binding of VLP affinity selectants to 4G2. ELISA plates were coated with 500ng of selected VLPs or rAMA1 and different amounts of 4G2 were applied. End-point dilution titers were determined as the reciprocal of the highest sera dilution with an OD greater than 2-fold higher than the no antibody control. The error bars represent the standard error of the mean of triplicate measurements.

We chose eight 4G2-selected VLPs from the mutagenic and the mixed libraries to test further. These included three VLPs from the mutagenic library and five VLPs from the random library that were representative of major sequence families. Plasmids encoding VLP selectants were constructed, verified by sequence analysis, and then used to generate VLPs. 4G2 binding to each of the VLPs was confirmed by end-point dilution ELISA (Fig 5). The three VLPs selected from the mutagenic library all bound strongly to 4G2, and slightly stronger (~10-fold) than the parental clone (NWDPIQFPGK) from which they were derived. In general, the VLPs from the mixed library selection also showed strong binding to 4G2, but two selectants from one family (VTHDGLEGQM and VTHDAWRPD) bound 100–1000 fold less than other selectant VLPs.

**Fig 6:**
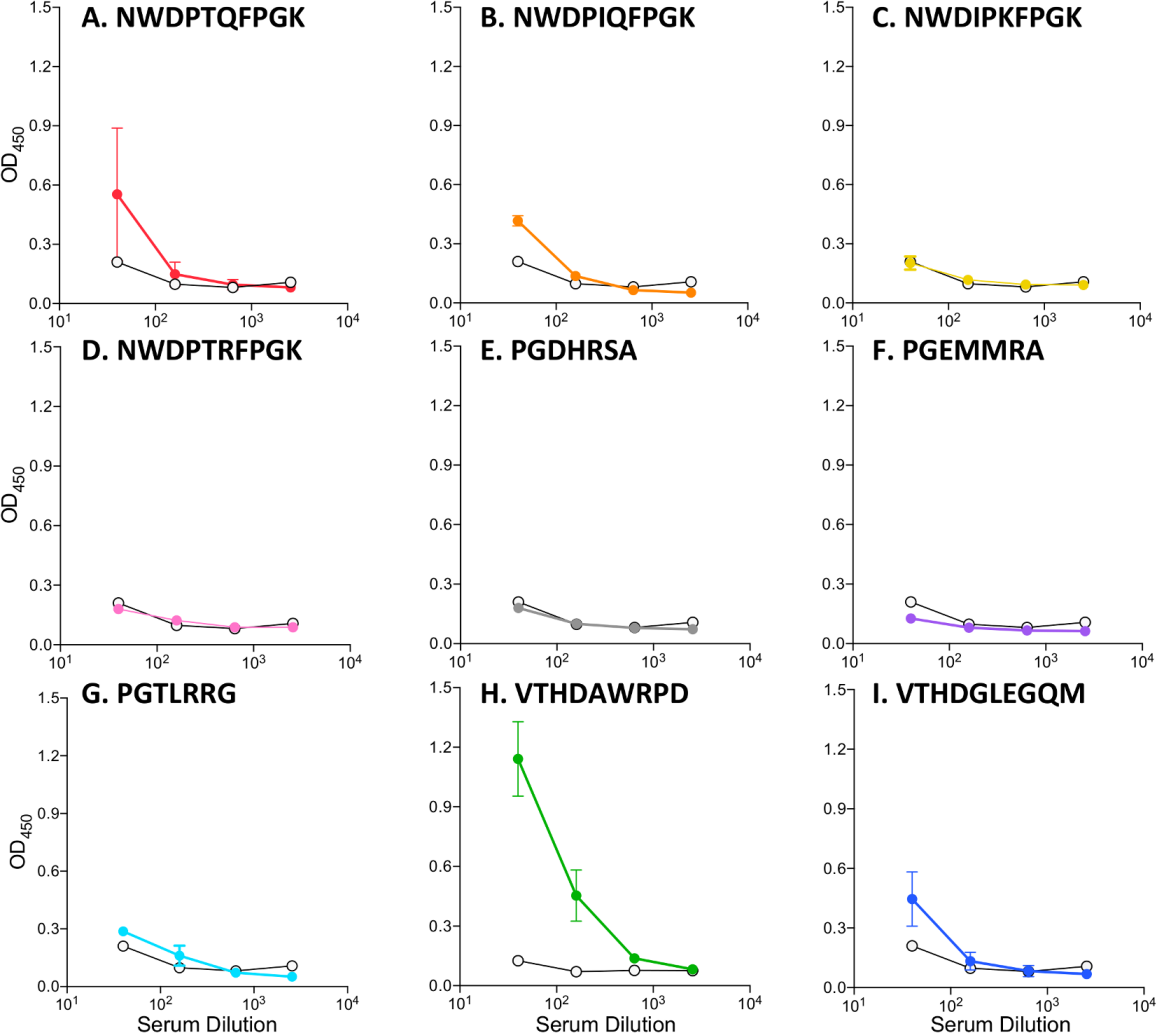
A subset of selectant VLPs elicit AMA1-reactive antibody responses. Groups of mice were immunized with VLPs displaying the listed peptide sequences. Each group consisted of three mice, except for group H, in which six mice were immunized. Anti-AMA1 IgG responses were measured by ELISA. Mean ODs and SEM are shown for each group.

Each of these VLPs was used to immunize a group of 3–6 Balb/C mice. Mice were immunized three times at two-week intervals with 10 μg of VLPs and sera were taken two weeks after the final boost. Anti-AMA1 antibody responses were measured by ELISA and compared to control mice that were immunized with wild-type MS2 VLPs (Fig 6). Most of the VLPs either failed to elicit AMA1-binding antibodies (panels C-G) or elicited antibodies that only weakly bound to AMA1 (panels B and I). However, surprisingly, the VLP that bound to 4G2 the weakest (MS2-VTHDAWRPD, panel H) elicited AMA1 cross-reactive IgG responses in all six mice that we tested.

**Fig 7:**
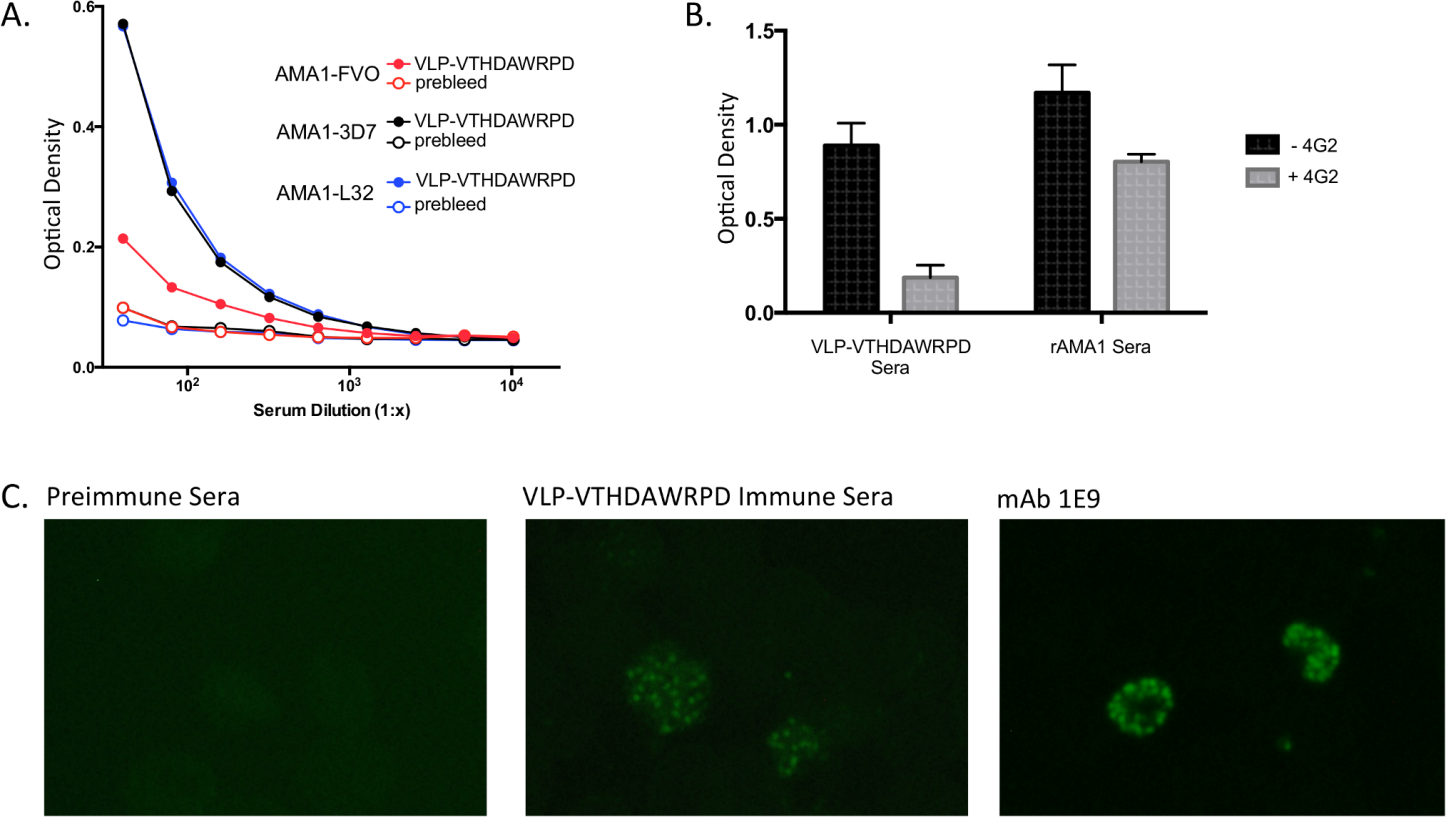
Sera from mice immunized with VLPs displaying the VTHDAWRPD peptide binds to the 4G2 epitope. (A) Reactivity of IgG in pooled sera from mice immunized with VLPs displaying the VTHDAWRPD peptide (closed circles) against recombinant AMA1 from 3D7, FVO, and L32 strains. Pooled preimmune serum (open circles) was used as a negative control. (B) Anti-AMA1 reactivity of pooled sera from mice immunized with VLPs displaying the VTHDAWRPD peptide (diluted 1:40) or AMA1 (diluted 1:1,000) in the presence (grey bars) or absence (black bars) of mAb 4G2. (C) Immunofluorescence. Immunosera binds to native AMA1 on merozoites. Slides were stained with pooled preimmune sera (1:25 dilution; left panel), pooled sera from mice immunized with VLP-VTHDAWRPD (1:25; middle panel), or, as a positive control, the anti-AMA1 mAb 1E9 (right panel).

ELISA binding data suggested that MS2-VTHDAWRPD VLPs elicited antibodies that bound to the 4G2 epitope on AMA1. To confirm this, we assessed the ability of these sera to bind to AMA1 from other *P*. *falciparum* strains, compete with 4G2 for binding to AMA1, and to bind to native parasite. Although AMA1 is polymorphic and has strain-specific variable regions, the 4G2 epitope is highly conserved between strains. We tested serum reactivity to AMA1 from three strains of *P*. *falciparum*, 3D7, FVO, and L32. The sequences of the FVO and L32 AMA1 antigens differ from 3D7 by 19 and 24 amino acids, respectively. However, none of these amino acid differences are located in the putative 4G2 binding region. As shown in Fig 7A, sera from vaccinated mice bound to AMA1-3D7, AMA1-FVO and, to a lesser extent, AMA1-L32. To discount the possibility that immunosera from VLP-immunized mice bound to a domain of AMA1 outside of the 4G2 epitope, we assessed that ability of 4G2 to block serum binding to AMA1. Serum binding to rAMA1 was measured by ELISA in the absence or presence of 4G2. As shown in Fig 7B, pretreatment of AMA1 with 4G2 substantially decreased the binding of IgG from MS2-VTHDAWRPD VLP immunized mice to AMA1, consistent with the hypothesis that this sera binds to the 4G2 epitope. As expected, pretreatment with 4G2 had only a modest effect on the binding of polyclonal sera from mice immunized with AMA1-3D7 to recombinant AMA1, since this sera presumably binds to multiple epitopes on the protein. Sera from MS2-VTHDAWRPD VLP-vaccinated mice failed to inhibit 4G2 binding to AMA1 (data not shown); presumably this is because the anti-AMA1 titers induced upon vaccination with VLPs were low. Next, to test whether these sera bound to native PfAMA1, we assessed binding to 3D7 merozoites by immunofluorescence. As shown in Fig 7C, serum from mice immunized with the VLP selectant showed a similar staining pattern on merozoites as a control anti-AMA1 mAb (1E9). Thus, taken together, these data support the contention that the VTHDAWRPD peptide, when displayed on an MS2 VLP, is an immunogenic mimic of the 4G2 epitope.

### Predicted structures of selectant VLPs

**Fig 8:**
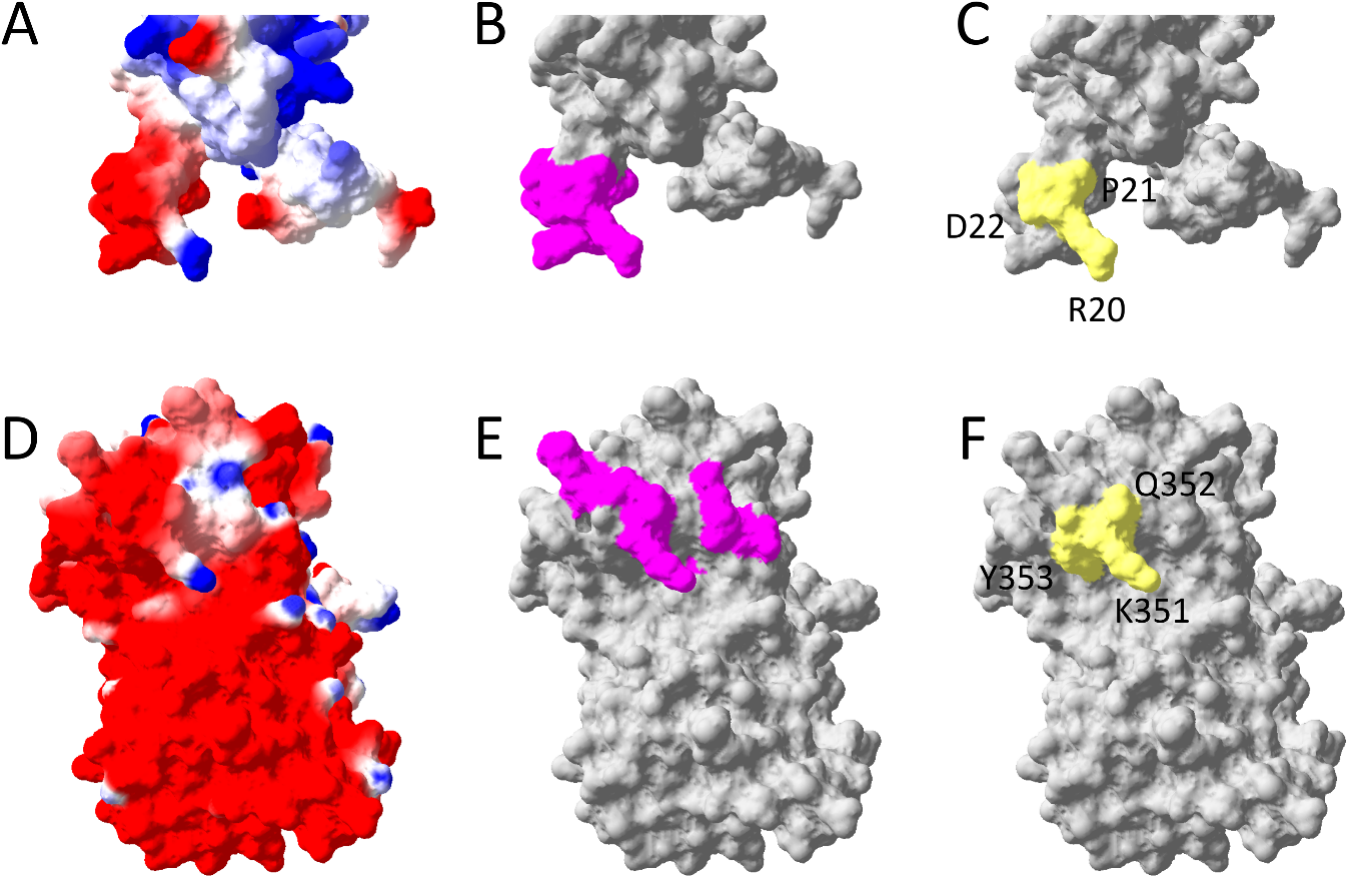
Comparison of the structure of the 4G2 epitope with the putative structure of the 4G2 mimotope on MS2 coat protein. (A) Predicted structure of the MS2-VTHDAWRPD coat protein dimer as determined by the One-to-One Threading function in the Web-based Phyre2 Server by modeling on MS2 coat protein (PDB: 1MSC). The VTHDAWRPD peptide, highlighted in panels B-D, is indicated by a dotted circle. Charge of proteins indicated by red (negative), blue (positive), and white (neutral). (B-D) A magnified view of the predicted structure of the VTHDAWRPD in the context of the MS2 AB-loop. (E-G) The structure of domains I and II of PfAMA1 (PDB: 1Z40_A). In panels (A,D) the charge of proteins indicated by red (negative), blue (positive), and white (neutral). Panel C highlights the VTHDAWRPD peptide insert and panel F shows a subset of the residues identified as necessary for 4G2 binding; both are indicated in magenta. In yellow space-fill, panel G shows selected residues on AMA1 involved in 4G2 binding (aa 351–353) and panel D shows putative residues that may be involved in 4G2 binding to VTHDAWRPD.

In order to investigate the structural basis of 4G2 mimics further, we generated predicted structural models of the selectant VLPs by using the Phyre2 Server. This web-based application can model protein structures based on sequence homology and predict structural features of user-entered protein sequences. For our purpose, we modeled the MS2 coat protein amino acid sequence with the foreign peptides against the structure for wild-type MS2 coat protein by using the One-to-One Threading function. This function allows modeling of the short constrained peptides in the AB-loop on the known structure of the MS2 coat protein dimer. Interestingly, the best immunologic mimic (VTHDAWRPD) had a striking similarity to a prominent feature of the AMA1 4G2 binding site (Fig 8), including a negatively charged “knuckle” (shown in red) and a positively charged “thumb” (shown in blue). Selectants that showed weak or no immunologic mimicry of the 4G2 epitope did not exhibit these features, although in some cases either a “knuckle” or a “thumb” feature was present (S1 Fig). These data suggest a possible explanation for the immunologic mimicry demonstrated by a subset of selectant VLPs.

## Discussion

Many pathogens have developed strategies to evade immunity by presenting epitopes to the immune system that can readily undergo antigenic variation while hiding highly conserved sites that are essential for protein function. Although it has often been difficult to target these conserved domains using vaccines, it has been increasingly possible to isolate monoclonal antibodies that target conserved epitopes and have broadly neutralizing activity. 4G2, for example, is a monoclonal antibody that recognizes a highly conserved epitope in the *Plasmodium falciparum* blood-stage antigen AMA1 and can potently inhibit parasite invasion of red blood cells [24]. 4G2 recognizes a discontinuous conformational epitope that is largely contained within the base of the domain II loop of AMA1 [11, 12, 25, 26]. Translating the detailed knowledge of the 4G2 epitope into a useful vaccine has been difficult, primarily because this epitope is poorly immunogenic in its native context. Nevertheless, presentation of this epitope in a sufficiently immunogenic form could yield an effective and broadly protective malaria vaccine.

Here, we targeted the 4G2 epitope using a novel VLP-based vaccine technology that allows for epitope discovery and mimicry on a highly immunogenic platform. Using three different selection strategies, we identified VLPs displaying different peptide sequences that bound strongly to 4G2. 4G2 recognizes a conformational epitope, so it was not surprising that our selections yielded VLPs displaying peptides that shared no obvious sequence identity to the residues that have been implicated in the 4G2 epitope on AMA1. It is well known, for example, that epitope mapping by filamentous phage display frequently finds peptides that bind the antibody at paratopes distinct from that bound by the antigen itself, and that this phenomenon is particularly common when using antibodies like 4G2 that do not recognize similar linear peptide epitopes [35–37]. Our data suggests that the majority of the peptides that we identified with our screen were so-called functional mimics; peptides that bound 4G2 at paratopes distinct from native AMA1. These selected VLPs did not elicit antibodies able to bind to AMA1.

In the initial 10mer selection, we isolated a VLP that bound strongly to 4G2, but only elicited AMA1 cross-reactive responses in about one-third of immunized mice. It is possible that this peptide shares some structural features with the native 4G2 epitope, but is a poor immunologic mimic. We addressed the question of whether our selectant could be improved as a mimotope by randomizing the VLP-displayed sequence and repeating affinity selection with 4G2. In this selection we also performed deep sequencing of the selected peptides at each round, and immunized mice with VLPs that were strongly enriched during the selection. One mutagenic selectant in particular (NWDPIQFPGK) was strongly enriched. Although antibodies elicited by this VLP bound only weakly to AMA1, all three immunized mice generated AMA1 cross-reactive antibodies.

These results were somewhat mirrored in the mixed library 4G2 selection. We sorted selectants into sequence families and investigated the immunogenicity of several of the most enriched or top-ranked family members. Although most of the selected VLPs did not elicit AMA1 reactive antibodies, despite strong binding to 4G2, we were able to identify a VLP selectant (MS2-VTHDAWRPD) that consistently elicited AMA1 cross-reactive antibodies. These sera bound to rAMA1 from three strains of *P*. *falciparum* by ELISA and to parasite by immunostaining. Interestingly, MS2-VTHDAWRPD VLPs bound quite weakly to 4G2, indicating that intrinsic reactivity to the selecting mAb does not necessarily predict immunogenicity. It is also noteworthy that VTHDAWRPD is a 9mer, which was not intentionally represented in the original mixed library. While the presence of occasional codon insertion or deletion errors aren’t surprising in the context of iterative amplification in affinity selection, it was interesting that a presumably rare mistake would have been selected for. A similar sequence (VTHDGLEGQM) also elicited AMA1-reactive antibodies, although the titers were much lower.

None of the peptides that we identified shared sequence homology to a set of 4G2 mimotopes that was previously identified by Casey and colleagues by filamentous phage display [31]. That study identified several peptides that bound strongly to 4G2; the two peptides that bound the strongest to 4G2 (J1 and J7) shared a common sequence motif (AFxDxxxVRxPxxY). These peptides also were immunogenic mimics. When used to immunize rabbits they elicited antibodies that bound to AMA1 and inhibited parasite invasion of erythrocytes. However, it’s unclear whether the antibody levels raised in animals were high enough to prevent invasion since the functional assays described in this manuscript were performed with IgG that was affinity-purified from sera using AMA1. While the basis of mimicry by the J1 and J7 peptides is not entirely clear, structural analysis revealed that the J1 peptide juxtaposes two amino acids motifs with homology to AMA1 that are also juxtaposed in the native structure of AMA1 [38].

It is likely that the diverse families of selectants that were identified by VLP display may bind to separate paratopes in the antigen-binding site of 4G2. In this way, selectant VLP families may be seen to have high affinity but may use separate molecular contacts to bind the mAb, and these would not act as mimotopes or elicit an antibody response similar to the native antigen. Our mixed library affinity selection data would suggest that it is important to investigate many families represented as selectants, even those that may exhibit limited binding to the selecting antibody such as the VTH group of sequences. The use of affinity selection coupled with deep sequencing facilitates this comprehensive analysis of selectant sequences. In addition, structural modeling could provide an additional tool for predicting and/or prioritizing possible immunologic mimics.

Although the two VLP selectants from the mutagenic and mixed library selections seem to be improved mimotopes, they elicited low titer anti-AMA1 antibody responses that are unlikely to be protective. However, it is possible that a weak 4G2-like response induced by a VLP-based vaccine could be boosted by vaccination with recombinant AMA1 protein. This prime-boost strategy might circumvent the issues of antigenic variation seen in the recent Phase 2 clinical trial utilizing a strain-specific AMA1 protein [21]. Recombinant AMA1 from almost any *Plasmodium* species or strain contains the conserved 4G2 epitope, and would presumably boost a pre-existing 4G2-like response similar to what we observed with the original group of 10mer-immunized responders [23]. This finding may have therapeutic implications as well. During the course of natural infection a 4G2-like response might be generated more rapidly if a pre-existing pool of partially cross-reactive B cells was available for boosting, targeting the immune response to the neutralizing (but normally immunosubdominant) 4G2 epitope [35]. The evidence that the 4G2 epitope is highly conserved indicates that it is not under strong immune pressure and/or is located in a critical region for parasite survival. Recent studies suggest that both mechanisms are probably responsible for conservation [11, 13]. With the proper stimulation however, B cells might more efficiently target the 4G2 epitope, and it is unlikely that AMA1 (and the parasite as a whole) could afford escape mutations at this site.

## Supporting Information

S1 Fig Predicted structure of the coat protein dimers of selected VLPs displaying 4G2 mimotopes. Predicted structures of the coat protein dimers were determined by the One-to-One Threading function in the Web-based Phyre2 Server by modeling on MS2 coat protein (PDB: 1MSC) and are shown grouped by ability to elicit AMA1 binding antibodies (A, best, B, weak, C, none). Each set of images shows the coat protein dimer from two angles. The prominent structure at the top of the dimer represents the AB-loop and the location of the mimotope peptide is denoted by an arrow in panel A. The charge of proteins indicated by red (negative), blue (positive), and white (neutral).
(TIFF)

S1 Table Verification of mutagenic plasmid library composition. Primers used for site-directed mutagenesis were designed by randomizing the nucleotide sequence encoding the 4G2 selectant NWDPTQFPGK (AAC TGG GAC CCG ACC CAG TTC CCC GGC AAG). Each of the thirty nucleotide positions in the mutagenic library was weighted for 76% chance of resembling the original nucleotide, or 8% chance of each other nucleotide. Percent occurrence of nucleotides was determined by deep sequencing of the plasmid library.
(DOCX)

S2 Table Top ~40 peptides from the each round of 4G2 affinity selection using a mutagenic library based on the peptide shown in the shaded row. Fold enrichment of peptides was calculated by dividing % total of the current round by the previous round % total. Coloring allows comparison of enrichment across the selection, and underlined peptides were further tested for immunogenicity.
(DOCX)

S3 Table Families of peptides from the 3^rd^ (final) round of 4G2 mixed-library affinity selection. Peptides in shaded boxes represent family consensus sequences, and bold peptides represent the highest-ranking members of each family (with the rank number noted). Underlined peptides were tested for immunogenicity. Bold sequences represent the highest ranked peptide within the family (based on total reads at the final round) and shaded cells contain the consensus sequence for each family.
(DOCX)

S4 Table The top selectants from each family of peptides after the 3^rd^ (final) round of affinity selection of the mixed-VLP library. The final rank number for each peptide is noted in parenthesis. Shaded rows highlight peptides tested for immunogenicity. Enrichment refers to the ratio of selectants with the given sequence (round 2:round 1 or round 3:round 2).
(DOCX)
