# Supplementary figures and images for: Identification of an Immunogenic Mimic of a Conserved Epitope on the Plasmodium falciparum Blood Stage Antigen AMA1 Using Virus-Like Particle (VLP) Peptide Display
Source: PLoS One. 2015 Jul 6;10(7):e0132560. doi: 10.1371/journal.pone.0132560 (PMC4493041; doi:10.1371/journal.pone.0132560)

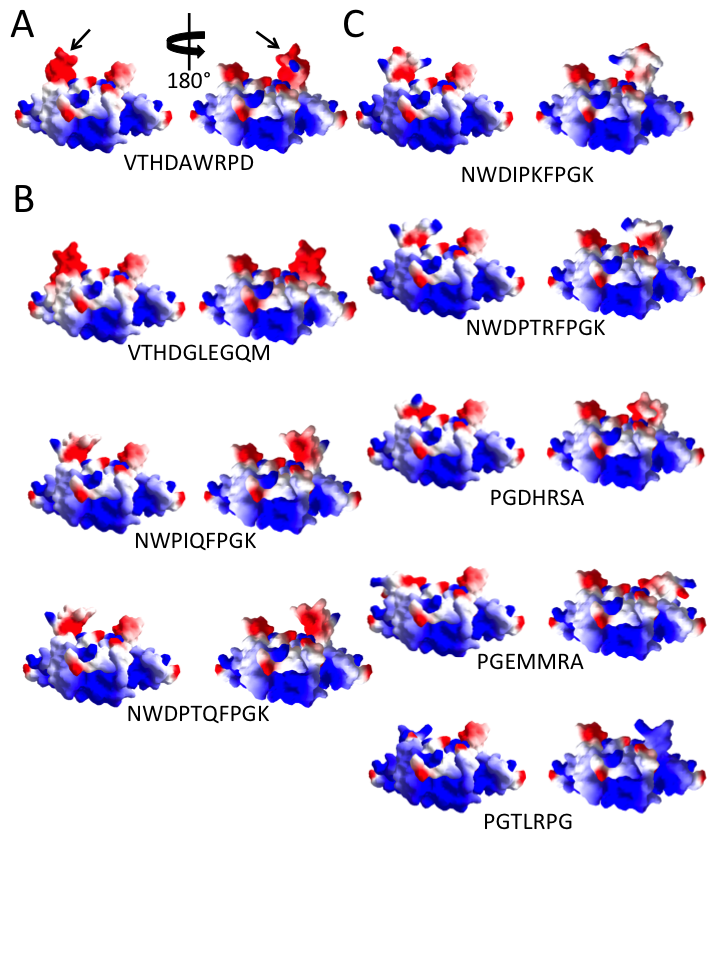

Supplement: S1 Fig — Predicted structures of the coat protein dimers were determined by the One-to-One Threading function in the Web-based Phyre2 Server by modeling on MS2 coat protein (PDB: 1MSC) and are shown grouped by ability to elicit AMA1 binding antibodies (A, best, B, weak, C, none). Each set of images shows the coat protein dimer from two angles. The prominent structure at the top of the dimer represents the AB-loop and the location of the mimotope peptide is denoted by an arrow in panel A. The charge of proteins indicated by red (negative), blue (positive), and white (neutral). (TIFF) [file pone.0132560.s001.tiff]
