# Supplementary material for: Identification of an Immunogenic Mimic of a Conserved Epitope on the Plasmodium falciparum Blood Stage Antigen AMA1 Using Virus-Like Particle (VLP) Peptide Display
Source: PLoS One. 2015 Jul 6;10(7):e0132560. doi: 10.1371/journal.pone.0132560 (PMC4493041; doi:10.1371/journal.pone.0132560)
Supplement: S1 Table — Primers used for site-directed mutagenesis were designed by randomizing the nucleotide sequence encoding the 4G2 selectant NWDPTQFPGK (AAC TGG GAC CCG ACC CAG TTC CCC GGC AAG). Each of the thirty nucleotide positions in the mutagenic library was weighted for 76% chance of resembling the original nucleotide, or 8% chance of each other nucleotide. Percent occurrence of nucleotides was determined by deep sequencing of the plasmid library. (DOCX) [file pone.0132560.s002.docx]

**Supplementary Table 1**. Verification of mutagenic plasmid library composition.

Primers used for site-directed mutagenesis were designed by randomizing the nucleotide sequence encoding the 4G2 selectant NWDPTQFPGK (AAC TGG GAC CCG ACC CAG TTC CCC GGC AAG). Each of the thirty nucleotide positions in the mutagenic library was weighted for 76% chance of resembling the original nucleotide, or 8% chance of each other nucleotide. Percent occurrence of nucleotides was determined by deep sequencing of the plasmid library.

| Nucleotide position | Percent occurrence of nucleotides at each position: | | | |
| --- | --- | --- | --- | --- |
|  | **A** | **G** | **C** | **T** |
| 1 (A) | 78.716 | 7.047 | 6.139 | 8.098 |
| 2 (A) | 78.768 | 7.612 | 6.360 | 7.260 |
| 3 (C) | 8.965 | 7.408 | 75.114 | 8.513 |
| 4 (T) | 9.081 | 7.037 | 8.014 | 75.868 |
| 5 (G) | 10.541 | 73.913 | 6.826 | 8.721 |
| 6 (G) | 10.177 | 73.874 | 6.927 | 9.022 |
