# Supplementary material for: Identification of an Immunogenic Mimic of a Conserved Epitope on the Plasmodium falciparum Blood Stage Antigen AMA1 Using Virus-Like Particle (VLP) Peptide Display
Source: PLoS One. 2015 Jul 6;10(7):e0132560. doi: 10.1371/journal.pone.0132560 (PMC4493041; doi:10.1371/journal.pone.0132560)
Supplement: S2 Table — Fold enrichment of peptides was calculated by dividing % total of the current round by the previous round % total. Coloring allows comparison of enrichment across the selection, and underlined peptides were further tested for immunogenicity. (DOCX) [file pone.0132560.s003.docx]

**Supplementary Table 2.** Top ~40 peptides from the each round of 4G2 affinity selection using a mutagenic library based on the peptide shown in the shaded row.

Fold enrichment of peptides was calculated by dividing % total of the current round by the previous round % total. Coloring allows comparison of enrichment across the selection, and underlined peptides were further tested for immunogenicity.

| **Original** mutagenic library | # of  reads | %  of total | **Round 1** peptide selectants | # of  reads | %  of total | Fold enrich-ment | **Round 2** peptide selectants | # of  reads | % of total | Fold enrich-ment |
| --- | --- | --- | --- | --- | --- | --- | --- | --- | --- | --- |
| NWDPTQFPGK | 99 | 0.105 | NWDPTQFPGK | 1511 | 2.168 | 20.66 | NWDPTQFPGK | 12115 | 8.180 | 77.98 |
| KWDPTQFPGK | 34 | 0.036 | **NWDPIQFPGK** | 890 | 1.277 | 86.06 | **NWDPIQFPGK** | 12049 | 8.136 | 548.39 |
| NWDPTQLPGK | 32 | 0.034 | **NWDPTQYPGK** | 766 | 1.099 | 69.13 | **NWDPIKFPGK** | 7310 | 4.936 | 132.34 |
| NRDPTQFPGK | 30 | 0.032 | NWDPTQFPDK | 681 | 0.977 |  | **NWDPTKFPGK** | 6599 | 4.456 | 210.24 |
| NWDPTQFPGN | 25 | 0.026 | **NWDPNQFPGK** | 664 | 0.953 | 56.18 | **NWDPNQFPGK** | 5653 | 3.817 | 4.01 |
| NWEPTQFPGK | 25 | 0.026 | **NWDPTKFPGK** | 605 | 0.868 | 40.95 | **NWDPAKFPGK** | 5505 | 3.717 | 5.88 |
| NLDPTQFPGK | 24 | 0.025 | **NWDPAKFPGK** | 441 | 0.633 | 597.0 | **NWDPTQYPGK** | 5340 | 3.606 | 3.28 |
| **NWDPTKFPGK** | 20 | 0.021 | KWGQTALPDM | 395 | 0.567 |  | NWDPVKFPGK | 4284 | 2.893 |  |
| NWDTTQFPGK | 19 | 0.020 | KWDATWFPVN | 392 | 0.562 |  | **NWDPTRFPGK** | 3684 | 2.487 | 4.88 |
| NCDPTQFPGK | 18 | 0.019 | NWNPTQFPGK | 369 | 0.529 | 27.75 | NWDPNKYPGK | 3266 | 2.205 |  |
| NWDPSQFPGK | 18 | 0.019 | NWDPTQFPGY | 368 | 0.528 |  | NWDPAQFPGK | 2962 | 2.000 |  |
| NWDPTQFPRK | 18 | 0.019 | NWDSTQFCGN | 361 | 0.518 |  | NWDPTKFPGM | 2286 | 1.544 |  |
| NWDPTQFSGK | 18 | 0.019 | **NWDPTRFPGK** | 355 | 0.509 | 43.69 | NWDPSQFPGK | 1988 | 1.342 |  |
| NWDPTQIPGK | 18 | 0.019 | NWKPIQLPGT | 339 | 0.486 |  | NWDPARYPGK | 1760 | 1.188 |  |
| NWNPTQFPGK | 18 | 0.019 | SQDPTQYSDK | 335 | 0.481 |  | NWDPTQYPGM | 1641 | 1.108 |  |
| NGDPTQFPGK | 17 | 0.018 | NSVPSQYSGK | 332 | 0.476 |  | NWDPAKYPGK | 1634 | 1.103 |  |
| NWDPTHFPGK | 17 | 0.018 | NWGPNKFPGK | 324 | 0.465 |  | NWDPTRYPGK | 1602 | 1.082 |  |
| NWDPTQFPGT | 17 | 0.018 | TWHPSQFHGM | 321 | 0.460 |  | NWDPKQFPGK | 1537 | 1.038 |  |
| NWDPTQVPGK | 17 | 0.018 | IYYQTTFSGY | 319 | 0.458 |  | NWDPIQFPGM | 1399 | 0.945 |  |
| NWDLTQFPGK | 16 | 0.017 | NWDPTQYPGL | 319 | 0.458 |  | NWDPSKFPGK | 1393 | 0.941 |  |
| **NWDPNQFPGK** | 16 | 0.017 | NWDPAQFPGK | 314 | 0.450 |  | NWDPTQFPGM | 1235 | 0.834 |  |
| YWDPTQFPGK | 16 | 0.017 | NWDPIQFRGK | 314 | 0.450 |  | NWDPIQFPGR | 1107 | 0.747 |  |
| NWDPTQSPGK | 15 | 0.016 | NWDPSQFPGK | 302 | 0.433 |  | NWDPIQYPGK | 1082 | 0.731 |  |
| **NWDPTQYPGK** | 15 | 0.016 | IGDPRQCSVQ | 295 | 0.423 |  | NWDPTQYPGL | 1028 | 0.694 |  |
| SWDPTQFPGK | 15 | 0.016 | NWNPTLYQYM | 292 | 0.419 |  | NWDPVQFPGK | 1013 | 0.684 |  |
| NSDPTQFPGK | 14 | 0.015 | HWADTQFHRT | 283 | 0.406 |  | NWDPNQFPGM | 997 | 0.673 |  |
| **NWDPIQFPGK** | 14 | 0.015 | NWDQNKFPGM | 281 | 0.403 |  | NWDPNQYPGK | 954 | 0.644 |  |
| NWDPTQFPDK | 14 | 0.015 | NWDPTQCTTN | 270 | 0.387 |  | NWDPTKYPGK | 914 | 0.617 |  |
| TWDPTQFPGK | 14 | 0.015 | HLDHTDLPSK | 259 | 0.372 |  | NWNPTQFPGK | 880 | 0.594 |  |
| IWDPTQFPGK | 13 | 0.014 | NWDTTQFPDK | 257 | 0.369 |  | NWDPSEFPGK | 870 | 0.587 |  |
| NWDPTLFPGK | 13 | 0.014 | NWDPTDIPGK | 249 | 0.357 |  | NWDPTQFPGQ | 751 | 0.507 |  |
| NWDPTQFPCK | 13 | 0.014 | NCVPIQLFRK | 247 | 0.354 |  | NWDPSQYPGQ | 632 | 0.427 |  |
| NWDRTQFPGK | 13 | 0.014 | TWDPPQVPGK | 247 | 0.354 |  | NWDPIKFPGY | 622 | 0.420 |  |
| NWDSTQFPGK | 13 | 0.014 | RGDQTQFLGK | 243 | 0.349 |  | NWDPDRFPGK | 604 | 0.408 |  |
| NWHPTQFPGK | 13 | 0.014 | NGNPTQVPCE | 223 | 0.320 |  | NWDPCQFPGK | 584 | 0.394 |  |
| HWDPTQFPGK | 12 | 0.013 | NRVPTKIHDR | 223 | 0.320 |  | NWDQNKFPGM | 584 | 0.394 |  |
| NWAPTQFPGK | 12 | 0.013 | NWDPTEYPCK | 220 | 0.316 |  | NWDPYKFPGK | 541 | 0.365 |  |
| NWDPTQFAGK | 11 | 0.012 | NWDPTQFPGM | 220 | 0.316 |  | NWDPRRFPGK | 515 | 0.348 |  |
| NWDPTQFLGK | 11 | 0.012 | TSDSTQCAGK | 220 | 0.316 |  | GWDPLKFPGK | 502 | 0.339 |  |
| … |  |  | … |  |  |  | … |  |  |  |
| **NWDPTRFPGK** | 11 | 0.012 | **NWDPIKFPGK** | 26 | 0.037 | 35.20 |  |  |  |  |
| **NWDPIKFPGK** | 1 | 0.001 |  |  |  |  |  |  |  |  |
| **NWDPAKFPGK** | 1 | 0.001 |  |  |  |  |  |  |  |  |
