# Supplementary material for: Identification of an Immunogenic Mimic of a Conserved Epitope on the Plasmodium falciparum Blood Stage Antigen AMA1 Using Virus-Like Particle (VLP) Peptide Display
Source: PLoS One. 2015 Jul 6;10(7):e0132560. doi: 10.1371/journal.pone.0132560 (PMC4493041; doi:10.1371/journal.pone.0132560)
Supplement: S3 Table — Peptides in shaded boxes represent family consensus sequences, and bold peptides represent the highest-ranking members of each family (with the rank number noted). Underlined peptides were tested for immunogenicity. Bold sequences represent the highest ranked peptide within the family (based on total reads at the final round) and shaded cells contain the consensus sequence for each family. (DOCX) [file pone.0132560.s004.docx]

**Supplementary Table 3.** Families of peptides from the 3^rd^ (final) round of 4G2 mixed-library affinity selection.

Peptides in shaded boxes represent family consensus sequences, and bold peptides represent the highest-ranking members of each family (with the rank number noted). Underlined peptides were tested for immunogenicity. Bold sequences represent the highest ranked peptide within the family (based on total reads at the final round) and shaded cells contain the consensus sequence for each family.

| **RVSRRGGP** | **GPGRMR** | **PGDHRSA** | **PGEMERA** | **IEHGPVA** | **PGHPRRG** | **VTHDALEGQM** | **ASAAGRA** |
| --- | --- | --- | --- | --- | --- | --- | --- |
| ALSAGGP | GAGQMR | HGDHRSA | PADDEAA | IEHEPVA | PGHLRRG | DHARRLEGQM | ASAAGGP |
| AVSAGGP | GAGRMR | LGDHRSA | PAYDEAA | **IEHGPVA #37** | PGHPRRG | VTARRLEGQM | ASAAGRP |
| HVSAGGP | GPGQMR | **PGDHRSA #1** | PGDDEAA | IEHGPAA | PGPLRRG | VTHDRLEGQM | ASAPGGP |
| RVSAGGP | GLGRMR | RGDHRSA | PGDDERA | IEHGPIA | **PGTLRRG #10** | VTHGRLEGQM | RSAAGGP |
| RVSAGG | GPGRMR | SGDHRSA | PGRDEAA | IEHGPVG | PRHLRRG | VTHDALEGQM | SSAAGGP |
| RVSAGGA | GQGRMR | TGDHRSA | PGADERA | IEHGPVR | PGHPARG | VTHDAWEGQM | ASAAGRA |
| RVSAGGPG | **GRGRMR #3** | PADHRSA | PGEDERA | IERGPVA | PRHPARG | VTHDGLEDQM | HSAAGRA |
| RVSAGGR | GSGRMR | PADHSSA | PGRDERA | IEHGPRR | PGTPRRG | **VTHDGLEGQM #91** | RASAAGRA |
| RVSAGRP | RAGRMR | PADTRSA | PGVDERA | IEHGAHR | PGTLRRGG | VTHDGLEGQT | **RSAAGRA #296** |
| RVSPGGP | GPGGMR | PAHHRSA | PGRDRAA | IEHGARR | PRYLRRG | VTHDGLGGQM | RSAPGGP |
| RVSRGGP | GPAGMR | PATHRSA | PGRHEAA |  | PGHPAAGG | VTHDRLGGQM | RSAPGRP |
| RVSRGG | GPGRMC | PAYHRSA | PAEDERA |  | PRHPAAGG | VTHDSLEGQM | RAQAAGRA |
| RVSAGRA | GPGRTR | PCDHRSA | PGEIERA |  | PRHPAAG | VTHGALEGQM | RARAAGRA |
| RVSAGSA | GPGRVR | PDDHRSA | PGEMERA |  |  | VTHGGLEGQM | RGSAAGRA |
| RVSAGRAR | GPSRMR | PRDHRSA | PGESERA |  |  | VTHNGLEGQM | RSAAGRAQ |
| RVSAGRV | HRYSGPGRMR | PSDHRSA | PGETERA |  |  | VTRDGLEGQM | RGRAAGRA |
| RVSRGRA | RPGRMR | PDNHRSA | PGEYERA |  |  | VTHDAWGGQM | RGRRAGRA |
| RVSRGRP | GRARMR | PGDDRSA | PGEIRRA |  |  | VTHDAWRGQM |  |
| RVSAGRGR | GRVRMR | PGDDRSP | PGEMEAA |  |  | VTHDAWEGPD |  |
| RVSRGRAR | GSDRMR | PGDDRSV | PGEMERP |  |  | VTHDALGGPD |  |
| RVSPGRGR | RAGTMR | PGDLRSA | PGEMGRA |  |  | VTHDGLEGPD | **PGKPRR** |
| RVSRGRGR | GRAGMR | PGDNRSA | PGEMKRA |  |  | VTHDAWRAQM | PGGPRR |
| RVSRGAGR | GSAGMR | PGDPRSA | PGEMMRA |  |  | VTHDAWRARW | PGGPRRSA |
| RVSRRRGR | GRAGCR | PGDQRSA | **PGEMRRA #2** |  |  | VTHDGLGGPD | PGKPRR |
| RVSRRRP | RPGTMR | PGDRRSA | PGEMSRA |  |  | **VTHDAWRPD #84** | **PGRPRR #324** |
| CVSRRAGP | GPAGCA | PGDTRSA | PGEMWRA |  |  |  | PGSPRR |
| CVSRRAGR | GPAGCC | PGDYRSA | RGESERA |  |  |  | RGGPRR |
| CVSRRGGP | GPAGCG | PGDHESA | PGETARA |  |  |  | PGVPRRSA |
| CVSRRVGP | GRAGCA | PGDHESP | PGETEAA |  |  |  | PGKPAR |
| HVSRRAGP |  | PGDHEVA | PGETERP |  |  |  | PGKAAR |
| RVSRRAGP |  | PGDHGSA | PGETRRA |  |  |  | PGKPAA |
| HVSRRAGR |  | PGDHKSA | PAEMRRA |  |  |  | PGRPAR |
| LVSRRAGR |  | PGDHMSA | PARMRRA |  |  |  | PGRPAA |
| **RVSRRAGR #4** |  | PGDHQSA | PATMRRA |  |  |  | RGRPRR |
| VSRRAGR |  | PGDHSSA | PDEMRRA |  |  |  | REGPRR |
| RVSRRGGP |  | PGDHWSA | PSEMRRA |  |  |  | PGKAAA |
| RVSRRVGP |  | PGDRESA | RAEMRRA |  |  |  | PGKARA |
| RASRRAGP |  | PGGHESA | PARLRRA |  |  |  | PGKPAAG |
| RASRRAGR |  | PGDTGSA | PARLERA |  |  |  |  |
| *(108 total in family)* |  | *(308 total in family)* | *(68 total in family)* |  |  |  |  |
