# Supplementary material for: Identification of an Immunogenic Mimic of a Conserved Epitope on the Plasmodium falciparum Blood Stage Antigen AMA1 Using Virus-Like Particle (VLP) Peptide Display
Source: PLoS One. 2015 Jul 6;10(7):e0132560. doi: 10.1371/journal.pone.0132560 (PMC4493041; doi:10.1371/journal.pone.0132560)
Supplement: S4 Table — The final rank number for each peptide is noted in parenthesis. Shaded rows highlight peptides tested for immunogenicity. Enrichment refers to the ratio of selectants with the given sequence (round 2:round 1 or round 3:round 2). (DOCX) [file pone.0132560.s005.docx]

**Supplemental Table 4.** The top selectants from each family of peptides after the 3^rd^ (final) round of affinity selection of the mixed-VLP library.

The final rank number for each peptide is noted in parenthesis. Shaded rows highlight peptides tested for immunogenicity. Enrichment refers to the ratio of selectants with the given sequence (round 2:round 1 or round 3:round 2).

| Mixed-library 4G2 selectants | Enrichment between rounds of selection: | |
| --- | --- | --- |
|  | **1^st^ – 2^nd^** | **2^nd^ – 3^rd^** |
| **PGDHRSA (1)** | 368.64 | 1.96 |
| **PGEMRRA (2)** | 421.57 | 1.01 |
| **GRGRMR (3)** | 42.70 | 0.34 |
| **RVSRRAGR (4)** | 203.08 | 0.25 |
| **PGTLRRG (10)** | 27.53 | 0.29 |
| **IEHGPVA (37)** | 1.63 | 0.49 |
| **VTHDAWRPD (84)** | 1.99 | 2.50 |
| **VTHDGLEGQM (91)** | 3.15 | 0.70 |
| **RSAAGRA (296)** | --- | 6.29 |
| **PGRPRR (324)** | 27.77 | 0.36 |
